# Recommendations for conducting qualitative research on multiple long-term conditions (MLTC) from a qualitative methods community of practice

**DOI:** 10.1177/26335565261459508

**Published:** 2026-06-10

**Authors:** Sue Bellass, Alison Ellwood, Stella Arakelyan, Sarah Pauline Bowers, Felicity Dewhurst, Stephanie J Hanley, Brenda Hayanga, Sarah A Hopkins, Nicola Howe, Emily Hunt, Grace Lewis, Kate Lippiett, Erin McCloskey, Sara Pretorius, Domna Salonen, Elizabeth Z Shoemark-Sells, Alexandra Thompson, Freya Thompson, Zoë Vowles, Heather Walker, Hannah Wheat

**Affiliations:** 1 AGE Research Group, Translational and Clinical Research Institute, Faculty of Medical Sciences, 5994 Newcastle University, Newcastle Upon Tyne, UK; 2 NIHR Newcastle Biomedical Research Centre, Newcastle Upon Tyne Hospitals NHS Foundation Trust, Tyne and Wear NHS Foundation Trust and Faculty of Medical Sciences, 5994 Newcastle University, Newcastle Upon Tyne, Northumberland, UK; 3 Academic Unit of Ageing and Stroke Research, 170791 Bradford Institute for Health Research, Bradford, UK; 4 Advanced Care Research Centre, Usher Institute, 215746 University of Edinburgh, Edinburgh, UK; 5 School of Medicine, 7486 University of St Andrews, St Andrews, UK; 6 Population Health Sciences Institute, 5994 Newcastle University, Newcastle Upon Tyne, UK; 7 Department of Applied Health Sciences, College of Medicine and Health, 1724 University of Birmingham, Birmingham, UK; 8 Department of Global Health and Social Medicine, Faculty of Social Science & Public Policy, 4616 King’s College London, London, UK; 9 Palliative and End of Life Care Group in Cambridge, Primary Care Unit, Department for Public Health and Primary Care, 2152 University of Cambridge, Cambridge, UK; 10 NIHR HealthTech Research Centre (HRC) in Diagnostic and Technology Evaluation, 5994 Newcastle University, Newcastle, UK; 11 Department of Sport, Health and Exercise Sciences, 3890 Brunel University of London, London, UK; 12 School of Health Sciences, 7423 University of Southampton, Southampton, UK; 13 Institute of Population Health, Faculty of Health and Life Sciences, 4591 Liverpool University, Liverpool, UK; 14 NIHR Newcastle Patient Safety Research Collaboration, 5994 Newcastle University, Newcastle, UK; 15 Faculty of Medicine, Health and Life Science, University of Swansea, Swansea, UK; 16 Guy’s and St Thomas’ NHS Foundation Trust/Department of Women and Children’s Health, School of Life Course and Population Sciences, Faculty of Life Sciences and Medicine, 4616 King’s College London, London, UK; 17 School of Cardiovascular and Metabolic Health, 3526 University of Glasgow, Glasgow, UK; 18 Community and Primary Care Research Centre, Peninsula Medical School (Faculty of Health), 6633 University of Plymouth, Plymouth, UK

**Keywords:** multiple long-term conditions, multimorbidity, qualitative research methods, community of practice, methods guidance

## Abstract

The growing prevalence of multiple long-term conditions (MLTC) – two or more long-term health conditions – has been recognised as one of the most significant health challenges facing contemporary societies. People living with MLTC experience poorer outcomes than people with one long-term condition and may be disadvantaged by health care systems configured for single conditions. Recognition of the significance of these inequities has led to increased investments in MLTC research, and a body of qualitative literature on lived experience is developing. However, conducting qualitative MLTC research presents challenges to researchers. Encompassing an extensive range of condition combinations, the MLTC population is incredibly diverse, and constructing a sampling strategy for the small numbers of participants typical in qualitative inquiry requires careful thought. Additionally, MLTC is a construct not embedded in public consciousness, which may affect participant self-identification and research engagement. Furthermore, the risk of issues commonly experienced in qualitative health research, such as participant distress and low recruitment rates, can be exacerbated due to the ill-health experienced by some people living with MLTC. In this article, we share reflections from a cross-institution Qualitative Methods Community of Practice in MLTC Research, describing the challenges experienced and practical steps taken to address difficulties and mitigate risks. We aim to provide tips and guidance to qualitative health researchers new to MLTC inquiry to support planning and delivery of their research in this rapidly growing field.

## Introduction

Multiple long-term conditions (MLTC) - two or more long-term health conditions^1^ - are becoming increasingly common worldwide and have been described as one of the major challenges facing contemporary medical science.^1–3^ Latest analyses in the UK state an overall prevalence of MLTC of 14.8%, rising to 68.2% in people aged 80 and over.^4^ Worldwide, evidence suggests 51.0% of people over 60 years of age live with MLTC.^5^

The consequences for people living with MLTC can be far-reaching. Compared to people with a single or no health condition, people with MLTC are more likely to experience lower healthy life expectancy, higher treatment and illness burden and poorer health outcomes.^6–8^ MLTC are associated with increased health resource utilisation including appointments and medication, yet people living with MLTC may be disadvantaged by a system that is orientated around single conditions and ill-equipped to respond to their needs.^9–11^ For instance, people living with MLTC can experience poorly co-ordinated care,^9^ conflicting advice from clinicians^12^ and problematic polypharmacy.^13^ In addition, the highly specialised nature of clinical education may reinforce disadvantage as clinicians may be guided to develop condition-specific skills over person-centred holistic approaches to care.^14^

Recognition of the accumulating impact of MLTC on individuals’ lives, and the challenge multiple conditions can present to healthcare services, has led to calls to prioritise MLTC research^1^ which have resulted in significant investments in UK research programmes.^15^ Understanding ‘what matters’^16^ to people living with MLTC, their carers and the care professionals who support them is a key component of this research endeavour. Qualitative health research is ideally positioned to generate this knowledge base due to its orientation towards understanding perspectives and identifying the contextual factors that inform service delivery and use. Conducting qualitative research in the MLTC space, however, is characterised by complexity. Qualitative approaches typically prioritise rich data from a relatively small sample of participants with shared characteristics,^17^ yet drawing qualitative samples from a heterogeneous population, when conditions may range from two well-managed non-impactful conditions through to debilitating conditions affecting several body systems,^18^ is complex. Further, as MLTC is a label used in clinical and research settings yet not in the wider public consciousness, recruitment can be challenging. In particular, potential participants may not self-identify as living with MLTC as they might with single diagnoses. These challenges, while widely experienced, have received minimal attention in published studies. In this paper we draw together the knowledge and experience of members of a cross-institution Qualitative Methods Community of Practice (CoP) in MLTC research established in 2022. First, we briefly describe the aims and objectives of the CoP before outlining some of the research challenges encountered by CoP members. We then offer recommendations to address those challenges to support successful planning and conduct of qualitative MLTC research.

## Qualitative methods community of practice in MLTC research

Navigating the early stages of a career in research, or being an experienced researcher embarking on a heterogeneous topic area such as MLTC, can be daunting and fraught with challenges, particularly in qualitative research, where limited understanding of qualitative approaches in research environments may increase the risk of researcher isolation and poor mental health.^19^ It is important, therefore, for qualitative MLTC researchers to have a peer support community that they can turn to for advice and signposting. Having opportunities for networking, the exchange of ideas and sharing of resources can facilitate the development of qualitative MLTC research skills, creating a robust foundation for the development of future research leaders.

In response to this need, and in the absence of existing MLTC-specific qualitative research networks, lead author SB established an online unfunded Qualitative Methods in MLTC Research Community of Practice (CoP) in December 2022. This was initially established with co-authors AE, SA, SJH and FT and formed part of the research capacity development workstream with the ADMISSION Research Collaborative.^20^ The membership and institutional reach have increased rapidly since its inception, currently standing at 28 early and mid-career researchers from 15 universities and National Health Service Trusts. Inspired by Wenger’s vision of learning as a social, rather than individual, process,^21^ the ethos of the CoP is to provide a supportive, informal environment where researchers can seek advice to address challenges other members may have encountered, in order to develop knowledge relevant to MLTC research. Four key objectives were developed in line with this aim:

1. To provide a forum to discuss methodological, ethical and practice aspects of qualitative research with people with MLTC and identify ways to address challenges experienced
2. To explore how both qualitative and quantitative inquiries can be incorporated in mixed-method studies
3. To develop a sustainable research community that provides opportunities to network and generate collaborative outputs
4. To share resources, ideas and best practice to build methodological skills and confidence within the community

The CoP membership spans a broad range of academic and clinical disciplines, methodological approaches and philosophical and theoretical perspectives to qualitative and mixed-methods MLTC research. Recognition of the value of our knowledge development has motivated members to share our learnings with the wider MLTC community, for while a body of empirical qualitative MLTC research is developing,^22–25^ the methodological complexities encountered during MLTC research remain under-reported. In this paper, we draw together our collective experiences of conducting research in this field to elucidate the challenges encountered and the solutions developed, with the aim of providing advice and guidance for novice MLTC qualitative researchers. We have endeavoured to sequence our guidance according to a typical qualitative research trajectory, outlining useful theoretical approaches, good practice for patient and public involvement and approaches to recruitment and data analysis, providing considerations for ethical approval applications throughout.

## Theoretical frameworks

Theory has an important role to play in framing qualitative MLTC research approaches due to the heterogeneity of the topic. The concept of MLTC is, at one and the same time, both useful and challenging. On one hand, focussing on MLTC brings attention to the deficits of a single-condition care model and foregrounds the poorer health outcomes and care navigation challenges experienced by people with MLTC. On the other, the concept of MLTC maintains a focus on diagnosed conditions and may overlook what really matters for health and wellbeing. One means of addressing this issue is to apply theoretical frameworks that view health and illness beyond a diagnostic lens, while focussing recruitment on MLTC populations. Use of such frameworks also shapes the overall research approach, feeding into research proposals, ethics applications and interview topic guides.

A broad range of theoretical approaches can underpin MLTC qualitative research, and it is beyond the scope of our paper to provide an exhaustive guide to the various approaches a researcher might choose. Instead, we outline briefly some potential frameworks from a range of disciplinary or philosophical positions that CoP members have found helpful and intriguing.

Biographical disruption^26^ and Burden of Treatment theory^27^ are examples of sociological perspectives on long-term illness that have been employed by our members. Biographical disruption, founded on the concept of chronic illness as a disruptive force that overturns taken for granted assumptions and introduces changes to one’s sense of self, orientation in the social world and distribution of resources, has the potential to offer insights into the numerous disruptions that multiple conditions might cause.^28^ Burden of Treatment theory^27^ draws attention to people’s agency and relationality, considering the activities people carry out to participate in healthcare services, maintain their quality of life and the social networks that they operate in. This theory foregrounds the dynamic nature of MLTC experiences, acknowledging that people living with MLTC likely need to continually review their situation and re-adjust their actions accordingly.^24^

Approaches using multi-level analysis include Bronfenbrenner’s ecological systems theory,^29^ Engel’s biopsychosocial model^30^ and critical theory.^31,32^ Bronfenbrenner’s theory is an example of a broad framework that can help to integrate thinking of complex adaptive systems into MLTC qualitative research by highlighting the interconnectedness of individual, social, and systemic factors affecting health experiences. By utilizing this framework, qualitative research may be able to generate actionable insights and recommendations to enhance integrated and compassionate healthcare solutions for individuals with MLTC. Engel’s biopsychosocial model encourages a holistic, person-centred approach to understanding MLTC, recognising the interactions between the biological, psychological and social domains.

Critical theory, an umbrella term for a range of standpoints, can bring clarity to understandings of MLTC experiences. Critical theory exposes power imbalance, social injustice, and causes of inequity. MLTC researchers exploring experiences of minoritised ethnic groups, for instance, could draw upon intersectionality, a theoretical framework that posits that social categories at the macrolevel (e.g., gender, class, race/ethnicity, disability, sexual orientation) cannot be understood in isolation.^31,32^ Instead, they intersect with macrolevel structural factors (i.e. sexism, classism, racism) resulting in disparate health outcomes which vary according to time and place.^31,32^ An intersectionality-informed approach can illuminate how ethnic inequalities in MLTC develop, are sustained and exacerbated, and how they can be addressed and prevented. Evidence from such an approach can inform the development of culturally appropriate solutions to adequately support minoritised ethnic people with MLTC to manage their conditions.

Realist evaluation^33^ and implementation models^34^ are approaches that focus on practice, understanding of implementation outcomes in context, and implementation evaluation. Implementation theories can be utilised in qualitative research to explore challenges to the embedding of new ways of working with and/or supporting those living with MLTC. The SELFIE framework,^35^ designed specifically for integrated care for MLTC, for example, provides useful guidance on implementation and evaluation. Normalisation Process Theory^36,37^ has been used to understand the processes by which practices do and do not become embedded in routine practice. This approach can then provide insight not only into what may present as less feasible or accessible components of a complex intervention for people with MLTC but also why these challenges to implementation may persist.

## Patient and public involvement (PPI) in MLTC research

Involving patients and members of the public throughout the study process from design to delivery is essential to ensure the relevance, acceptability and meaningfulness of research to people who will be affected by its outcomes.^16,38^ In the context of MLTC, which has higher prevalence in underserved groups, we advise researchers to develop a strategy to support the involvement of a diverse PPI group comprising people living with MLTC and carers from a broad range of backgrounds. However, the illness and treatment burdens that can be experienced by contributors mean that careful consideration needs to be given to facilitate diverse and inclusive PPI in MLTC research, for example, ensuring there are a range of ways to contribute to projects.

Co-author SJH has led a CoP focused on training for early-career researchers on best practice PPI with diverse populations. To foster inclusive PPI, MLTC research teams are encouraged to engage early with organisations in the voluntary, community, faith and social enterprise (VCFSE) sector. Such organisations play a critical role in facilitating access to, and building trust with, communities that are historically less represented in MLTC research and health research more broadly. Developing and sustaining collaborative relationships with VCFSE organisations requires dedicated time and resources but is essential for supporting meaningful and continuous involvement. In addition, collaboration with charity partners in the planning and delivery of public engagement events can facilitate the identification and inclusion of a diverse range of individuals from local communities.

## Sampling and recruitment strategies

### Representing MLTC

In the qualitative research paradigm we do not typically aim for a representative sample from which findings can be generalised^39,40^; nevertheless we wish to construct samples that enable generation of salient knowledge of a particular phenomenon. This is challenging within qualitative MLTC research, where the lived experience of well-managed conditions may be very different to those of severe conditions which have a significant and limiting impact on daily life. The concept of ‘complex MLTC’ has been used by some researchers as a framing device, though there is no single accepted definition,^41^ and it may not be considered as an acceptable term by some people living with MLTC to describe their experience. When exploring the lived experience of MLTC, it is common to stipulate two or more long-term medical diagnoses of any kind as the eligibility criteria to take part in the research. Given that the inclusion of people living with long-term conditions is notoriously challenging,^42,43^ having two or more conditions as the sole criterion can potentially lead to an unbalanced sample where people who are more unwell are under-represented. In one of our studies, for instance, 19.6% (18/92) of potential participants who had expressed an interest in taking part ultimately declined due to ill-health.^44^ Conversely, those with well-managed MLTC may report little or no consequences of their conditions, in which case varied methods may be required to explore diverse experiences.

Reflecting the entire spectrum of MLTC experiences in qualitative research appears unrealistic given the small samples typically recruited. Instead, at the outset of their research, qualitative MLTC researchers need to think carefully about the contribution they aim to make to the field and how their recruitment strategy will ultimately lead to the generation of data to address the research questions. To ensure representation of a range of experiences, or to focus investigation at either end of the MLTC spectrum, requesting further information about diagnoses before conducting data collection may need to form part of the recruitment strategy.

In addition to the health conditions they live with, potential study participants are a heterogenous population in terms of their demographic characteristics i.e., age, gender, ethnicity, religious beliefs, socioeconomic status, sexual orientation and rurality. Evidence indicates that MLTC impact population groups differently,^45,46^ highlighting the need for diverse recruitment across various social contexts to ensure that the voices and experiences of marginalised populations are represented.^22,47^ An important additional consideration for researchers is that while MLTC are commonly associated with increased age, people living in deprived areas are likely to develop MLTC 10-15 years earlier,^48^ and MLTC emerge from midlife for many minoritised ethnic groups.^46^ Therefore, depending on the aims of the research, it may be important to plan to recruit younger cohorts, particularly if the research aims to explore inequities in MLTC.

### Identifying as eligible for MLTC research

While ethical challenges are encountered in all qualitative research,^49^ members of the CoP have encountered issues specific to MLTC research. Firstly, while providing clear, unambiguous information about research studies and participant eligibility is viewed as central to good ethical practice,^50^ the term ‘multiple long-term conditions’ is currently confined to research, clinical and health policy spheres. As MLTC is not yet embedded in public discourse, there is no accompanying social narrative which shapes public understanding as there is for common single health conditions such as diabetes, depression and heart disease. This has implications where research designs may require potential participants with lived experience of MLTC to self-present to studies. Participants may be uncertain which and how many conditions are included within the scope of MLTC, what counts as ‘long-term’, and whether remitting conditions or long-term symptoms without diagnoses are included. Listing health conditions as examples in promotional materials may inadvertently lead to people self-excluding. Further, liaising with professionals to identify potential research participants may result in individuals being approached for involvement in research when they do not identify as someone living with MLTC, which may not only pose a barrier to research engagement but also potentially cause confusion and distress.

When drafting the recruitment strategy for the application for ethical approval there are certain practices which may prove beneficial. In relation to recruiting participants, having clear participant inclusion and exclusion criteria and highlighting potential self-identification issues in advance with recruiting staff is helpful. Further, involving patient and/or public contributors in the development of recruitment strategies and promotional materials is an essential and important inclusive practice. We would also advise developing a clear plan in advance on how to respond if potential participants do not self-identify with MLTC. This could include, for instance, creating a distress protocol or sign-posting to a healthcare professional who can discuss their health conditions.

If it is necessary to create a list of conditions as examples in participant-facing documentation, we would suggest including examples of conditions affecting different body systems, congenital and acquired conditions, common conditions, mental health conditions and long-term infectious disease conditions. An example of such a list can be found on page one of a directive used in one of our studies at https://massobs.org.uk/wp-content/uploads/2024/07/Autumn_-FINAL_141123-1.pdf.

### Working with clinical specialties: Recruiting patient participants

Several of the CoP members liaised with healthcare professionals to recruit patient participants. In the context of MLTC, there may be additional issues researchers need to consider. For example, care for people living with MLTC is often centred around a single health condition,^3,51^ typically the one which requires the highest level of clinical input. As such, this creates challenges for clinicians when screening clinic lists to identify people living with MLTC to participate in research. Although most of our experience relates to secondary care settings, we would anticipate that similar issues may be encountered in recruitment through primary care. Here, we would like to highlight a best practice approach identified by two of our CoP members (Case Study 1).

**Case study 1: MuM-PreDiCT: Working with clinicians to develop a successful recruitment strategy**

The MuM-PreDiCT project is focused on understanding and improving maternity care for women with MLTC.^52^ Authors SJH and ZV were involved with the qualitative work package and SJH coordinated the recruitment of 57 pregnant and postnatal women living with MLTC. Five NHS sites supported recruitment. In the early months, having sought feedback from research midwives who found recruiting diagnosis-agnostic MLTC challenging, it became apparent that a focused recruitment approach was required. As such, in alignment with the key conditions identified in recent MBRRACE-UK maternal mortality reports,^53^ the multidisciplinary project team created a revised recruitment strategy whereby cardiac conditions were first prioritised and through continuous reflection of the sample characteristics and informed by wider MuM-PreDiCT epidemiological work,^54^ the approach was adapted as recruitment progressed. Regular discussions were held between the clinical research midwives and the study team regarding ongoing priorities for recruitment to the study. This enabled the clinical research midwives to communicate regularly with clinical colleagues including midwives, obstetricians and obstetric physicians regarding study recruitment. This facilitated identification of women who were eligible for the study and met the iterative sampling criteria.

### Non-NHS recruitment strategies

#### Recruiting through voluntary sector organisations

Identifying and building trusting relationships with community groups and voluntary sector organisations provides a valuable alternative or complement to traditional NHS facilitated recruitment methods for engaging with a diverse range of MLTC communities. However, while organisations for single conditions exist, such as Dementia UK or the Motor Neurone Disease Association, there is currently no large-scale voluntary sector organisation in the UK specifically serving MLTC populations. Identifying single-condition groups with the aim of recruiting people with comorbid conditions may be one route to establishing a recruitment channel.

From the experience of our members, fostering connections with community groups at a very early stage in the research process, and ensuring there is time and funding available within the project to commit to this activity, is crucial. Involving community groups at the earliest stages allows for collaborative input on recruitment strategies and ensures feedback can refine and tailor approaches to the specific needs of the communities. Transparent reporting of findings back to communities reinforces the value of their contributions throughout the research and helps maintain long-term relationships for future studies.

#### Recruiting via social media: The problem of inauthentic participation

Some CoP members have attempted to recruit a diverse group of participants by advertising MLTC studies on social media. This allows researchers to reach potential participants who may not be identified by healthcare professionals or may have little or no contact with healthcare services. However, members of the CoP have had such efforts hindered by the challenge of inauthentic participation, where fraudulent individuals express interest in research participation despite not being eligible, leading to serious implications for research integrity.^55,56^ Remuneration seems to increase the risk of this phenomenon, and the time needed to robustly screen potential participants when recruiting via social media needs to be factored into project plans. While inauthentic participation is by no means unique to qualitative MLTC studies, researchers in this field may be drawn to social media recruitment due to recruitment from clinical sites potentially being complicated by lack of diagnostic specificity.

Prompt detailed screening of potential participants is crucial to allow researchers to distinguish between authentic and inauthentic participants. CoP members have observed that emails from inauthentic participants often appear from free email account platforms and appear as a combination of a name and a randomly generated number. The contents of emails often paraphrase the study advert entirely and provide little additional personal or clinical information. When additional information is sought through screening, this often can be inaccurate or incongruous to the clinical scenario. Potentially inauthentic participants generally request an online interview, in place of a face-to-face interview, and tend to reply extremely quickly initially, but there are often delays encountered with subsequent requests for more information. Mistry et al. (2024)^57^ advise conducting screening in advance of data collection, and provide detailed recommendations on how to confirm participant eligibility. They emphasise that screening questions should be carefully chosen to align with information governance frameworks and should be reviewed by the relevant research ethics committee. Incorporating various methods of recruitment within the study design can also aid in mitigating the risk of recruiting inauthentic participants.

## Experiences of data collection

Many of the CoP members have used semi-structured interviews and focus groups, established qualitative research methods,^17^ while others have found creative methods such as digital storytelling^58^ to be particularly helpful. Vignettes can also be an effective approach to gain insights into MLTC experiences. Here, we describe a study (Case Study 2) engaging healthcare professionals as study participants, where MLTC vignettes were created to explore clinical decision-making processes. We outline the decisions made and the opportunities that this method presents to understand provision of care for people living with MLTC.

**Case study 2: ADMISSION-CPA: Using clinical vignettes to elicit data on clinical decision-making in the context of MLTC**

The use of clinical vignettes (hypothetical patient scenarios that outline specific health and social contexts)^59^ in MLTC research can be an effective tool to stimulate engagement from clinical specialists and to demonstrate relevance to real-world settings and clinical practice.^60^ In ADMISSION-CPA,^20^ a study of clinical decision-making for people with MLTC admitted to hospital conducted by authors SP and NH within a wider team, five distinct clinical scenarios were devised describing patients presenting with a range of condition combinations and including details on social and behavioural factors. The vignettes were deliberately designed to have no ‘right answer’ to enable decision-making processes to be interrogated. We piloted the vignettes and considered how best to align the vignettes with participants’ expertise, remaining flexible in interview scenarios and presenting alternative vignettes when necessary. We found that participants often referred to the patients described in the vignettes as though they were real individuals, suggesting that the scenarios resonated with their clinical experience. In some instances, participants used the vignettes not only to respond to vignette-specific prompts, but also as concrete examples when answering broader questions about the challenges of decision-making for people living with MLTC. This blurring between hypothetical and real-world contexts highlights the potential of clinical vignettes to anchor abstract discussions in lived clinical realities but also requires careful interpretation during analysis. We had to decide whether to treat interviewee responses to vignettes as a continuation of the main body of the interview or analyse them separately, eventually opting for the latter. Because vignettes allow elicitation of interviewees thought processes, actions and external processes affecting these in practical situations, the data lend themselves to application of a theoretical framework such as The Theoretical Domains Framework, the COM-B system or ‘behaviour change wheel’.^61^

### Participant and researcher distress

Living with MLTC has been linked to numerous adverse health outcomes and reduced quality of life. Alongside this, the challenge of navigating care systems can lead people to become distressed and disillusioned about the care they receive, which they may express to researchers. While participant distress can occur in any study, the risk may be higher in MLTC research where people can be experiencing high levels of illness and treatment burdens. In addition, gathering stories about negative experiences and difficulties faced by participants can have an emotional impact on the researcher. We would, therefore, recommend the development of both participant and researcher safety protocols (e.g. the Qualitative Research Distress Protocol^62^), which include agreed plans on how to respond to a range of distress scenarios. Training and support may be needed for both researchers and supervisors or principal investigators to identify and manage distress.^62,63^

## Data analysis

The heterogeneity of qualitative MLTC data poses challenges that may not be encountered in single health condition research, where the analytic focus is likely to be on the experience of living with or caring for people with a defined set of symptoms. In contrast, with the plurality of conditions that MLTC encompasses, there are a range of possible directions an analysis could take, depending on the research questions underpinning the study. For instance, one option may be to compare and contrast experiences of concordant and discordant MLTC, i.e. conditions that share a pathophysiology and treatment plan or those that are unrelated and may have separate and even contradicting care plans. Another possibility might be to explore differences in mental, physical and long-term infectious conditions. However, such approaches are still framed biomedically. Instead, qualitative MLTC researchers may consider a psychosocial approach, classifying MLTC experiences according to different dimensions. These could include type of onset (sudden or gradual), course (progressive, constant or relapsing) and outcomes (life-limiting or life-shortening).^64^ Other options include taking a life course approach, exploring conditions at the time in which they emerge in people’s lives,^65^ using a chronic crisis approach to explore MLTC and social exclusion^66^ or looking at barriers to care for people with various MLTC combinations. Researchers may also have to grapple with the extent to which their findings are due to MLTC, or whether they are ‘every patient’ experiences, if the focus of the research is the setting in which care is provided rather than other aspects of MLTC experiences.

## Concluding thoughts and recommendations

This article describes the reflections of researchers within a community of practice conducting qualitative MLTC research. We would like to conclude by summarising our key recommendations for qualitative health researchers entering the burgeoning field of MLTC research for the first time. In doing so, we acknowledge that qualitative MLTC research is both challenging, and in relatively early stages, and that we cannot identify and resolve every challenge that qualitative MLTC researchers may encounter. Nevertheless, we hope to provide some guidance for those setting out in this exciting, vibrant and flourishing area of research. Based on our collective experience, we offer the following tips for those embarking on qualitative MLTC research:

• Identify a mentor with expertise in qualitative methods and/or join a qualitative methods community for peer support and sharing of best practice
• Explore the broad range of theoretical frameworks that can frame qualitative MLTC research, including biographical disruption, Burden of Treatment Theory, Normalisation Process Theory, Bronfenbrenner’s ecological systems theory, SELFIE framework for integrated care for MLTC and intersectionality
• Be aware that people living with MLTC may not identify with the term, and this may affect both self-presentation rates and/or recruitment via organisations. Discuss and agree recruitment strategies with health and social care professionals and build in adequate time for recruitment and engage public contributors in the development of participant-facing materials
• Formulate a recruitment strategy in the funding application that allows sufficient time and budget to recruit a diverse MLTC PPI group, build relationships with the VCFSE sector and manage any inauthentic participation encountered.
• Consider carefully how you will recruit your sample to align with your research questions. Being able to fully reflect the heterogeneity of MLTC in a relatively small qualitative sample is likely to be unrealistic; therefore, identify what you aim to contribute to the knowledge base, and what aspects of MLTC or populations living with MLTC you wish to focus on
• Prepare safety protocols and establish supportive processes with senior researchers in advance of undertaking fieldwork, given that illness/treatment burdens and complicated care pathways experienced by people living with MLTC may increase the risk of participant and researcher distress

## Data Availability

This is a methods paper rather than a findings paper. We are not reporting empirical data.
